# The use of ELISA is comparable to immunoprecipitation in the detection of selected myositis-specific autoantibodies in a European population

**DOI:** 10.3389/fimmu.2022.975939

**Published:** 2022-09-08

**Authors:** Aravinthan Loganathan, Fionnuala McMorrow, Hui Lu, Danyang Li, Ben Mulhearn, Neil John McHugh, Sarah Louise Tansley

**Affiliations:** ^1^ Royal National Hospital for Rheumatic Diseases, Bath, United Kingdom; ^2^ Department and Pharmacy and Pharmacology, University of Bath, Bath, United Kingdom

**Keywords:** inflammatory myositis, ELISA - enzyme-linked immunosorbent assay, immunoprecipitation (IP), myositis autoantibody, diagnostic test, diagnostic accuracy

## Abstract

**Background:**

The reliable detection of myositis-specific autoantibodies (MSA) provides valuable clinical information regarding prognosis, clinical progression and diagnostic confirmation.

**Objectives:**

To evaluate the reliability of a commercial ELISA immunoassay in detecting myositis-specific autoantibodies in comparison to immunoprecipitation as the reference standard.

**Methods:**

Serum samples were chosen from a biobank of more than 3000 samples. Samples with a confirmed MSA on Immunoprecipitation (n=116) were evaluated in duplicate by ELISA to detect Mi2, MDA5, Jo1, EJ, KS, PL-7 and PL-12 (Medical & Biological Laboratories Co. Ltd, Nagoya, Aichi, Japan). Healthy control samples (n=246) confirmed autoantibody negative by immunoprecipitation were similarly assessed.

**Results:**

There was a very good agreement between ELISA and immunoprecipitation for serum samples containing anti-Mi2, MDA5, Jo1, EJ, KS and PL-7 and PL-12 auto-antibodies. Cohen’s κ values ranged from 0.86-1 for the measured autoantibodies on ELISA.

**Conclusion:**

ELISA was an accurate method for detecting anti-synthetase, anti-Mi2 and anti-MDA5 autoantibodies.

## Introduction

Idiopathic inflammatory myositis (IIM) is a heterogeneous group of disease that can affect muscles, skin, and lungs. Confirmation of myositis-specific autoantibodies (MSAs) has important implications when diagnosing IIM in patients with high pre-test probability (1). Individuals diagnosed with IIM typically generate one type of MSA. Detection of specific MSA antibodies can guide clinicians to the disease course of a patient’s subtype of myositis and clinical outcomes (1, 2).

The presence of anti-MDA5 autoantibodies is associated with rapidly progressive interstitial lung disease (ILD), dermatomyositis (DM) associated skin rashes and cutaneous ulceration. Patients typically experience amyopathic or mild muscle disease. In comparison, anti-aminoacyl tRNA synthetase (ARS) antibodies (including Jo1, EJ, KS, PL-7 and PL-12) suggest an increased risk of developing significant interstitial lung disease with the presence of DM-associated skin rashes, Raynaud’s phenomenon, inflammatory arthritis, mechanics hand and fever. Mi2 autoantibodies suggest the presence of classical dermatomyositis rash with mild myositis and varying associations of malignancy reported in the literature (1, 2).

Immunoprecipitation is widely considered the reference standard for the detection of myositis autoantibodies. However, the cost, time, and requirement of specialist facilities and staff with expertise in performing and interpreting immunoprecipitation results have limited its widespread use (3). Utilisation of commercially available immunoassays such as ELISA and immunoblot has increased, but concerns regarding the sensitivity and specificity of these immunoassays in the real world exist (3–5). Serological screening using ELISA is a highly sensitive method for detecting the presence of an MSA; but requires a highly purified recombinant protein. Poor recombinant protein purification, protein expression, and lack of stability increase the risk of false-positive detection of autoantibodies when using ELISA (6). In Japanese cohorts, the sensitivity and specificity of detecting ARS antibodies (PL-12, PL-7, EJ, Jo-1 and KS) when using ELISA compared to IP is 97.1% and 99.1%, respectively (7). We have previously reported ELISA as an accurate test for detecting anti-TIF1γ with superior sensitivity to blotting-based assays (8).

This study aimed to compare the reliability of a commercial ELISA in detecting MSA’s compared to immunoprecipitation, the reference standard, in a European population. MSA’s included were anti-Mi2, MDA5, Jo1, EJ, KS, PL-7 and PL-12 from a cohort of adult patients confirmed to have the presence of an MSA on immunoprecipitation. A secondary outcome of this study determined whether using different cut-off points may improve the sensitivity and specificity of the immunoassay in a European population compared to a healthy control population.

## Methods

### Sample selection

Our laboratory has to date, analysed more than 3000 myositis serum samples by immunoprecipitation (2, 9). Patients included in this series were all identified through autoantibody analysis in our laboratory for research or diagnostic purposes. All had been previously screened for autoantibodies by immunoprecipitation, and samples for inclusion were randomly selected from each autoantibody subgroup. Healthy control (HC) samples had no known underlying rheumatological condition and were confirmed to be autoantibody negative by immunoprecipitation. The same serum sample was used for both immunoprecipitation and ELISA analysis. **Table 1** illustrates the number of sera samples with confirmed MSA’s and healthy controls used in this study.

**Table 1:** Number of sera samples tested on ELISA for both case samples and health controls (HC) and the number of samples with a co-efficient variance of equal to or less than 20%.

Autoantibody	^+^MSA Sample Number	HC Sample Number	^+^MSA sample with ^++^CV ≤20%	HC sample with ^++^CV ≤20%	Pre-defined positive cut-off (au)
Mi2	20	63	19	63	53
MDA5	20	63	20	62	32
ARS^*^ MSA	76	120	70	100	25
Jo1	22	66	21	61	25
EJ	10	54	7	50	25
KS	3	47	2	47	25
PL-7	21	65	21	60	25
PL-12	20	64	19	58	25

^*^Aminoacyl tRNA synthetase myositis specific antibodies combined (Jo1, EJ, KS, PL7, PL12).

^+^Myositis specific antibody samples.

^++^Co-efficient of variation.

### ELISA

ELISA was performed per the manufacturer’s instructions (Medical & Biological Laboratories Co. Ltd, Nagoya, Aichi, Japan). Briefly, 5µL of serum sample was used, and all samples were run in duplicate. Samples were thawed, diluted to a 1:101 concentration, and incubated on a microwell plate for 30 minutes. Samples were then incubated with a horseradish peroxidase conjugated goat anti-human IgG antibody conjugate for 30 minutes, followed by a TMB peroxides for 15 minutes. Reactions were terminated by 0.25 mol/L of sulfuric acid. These assays were performed at room temperature with four wash cycles between steps. The absorbance of each well was read on the FLUOstar Omega microplate reader (BMG Labtech Ltd., Aylesbury, Buckinghamshire, Great Britain) at 450 nm wavelength. Pre-defined positive cut-off values were 53 au for Mi2 (10), 32 au for MDA5 (11) and 25 au for ARS antibodies (Jo1, EJ, KS, PL-7 and PL-12) (7). Samples were tested in duplicate, and those with a coefficient of variation (CV) of greater than 20% were excluded from the final analysis (9). This assay does not differentiate between ARS antibodies. The interpretation of each specific ARS antibody depended on the initial immunoprecipitation result.

### Immunoprecipitation

Protein immunoprecipitation was performed and has previously been described by Tansley et al. (3). Briefly, sera (10µl) was mixed with 2 mg protein-A-Sepharose beads at room temperature for 30 minutes. Beads were washed in immunoprecipitation buffer before adding 120 ll (35S)methionine-labelled K562 cell extract. Samples were mixed at 4°C for two hours. Beads were washed in immunoprecipitation buffer and Tris-buffered saline before resuspending in 50 ll SDS sample buffer. After heating, proteins were fractionated by 9% SDS-PAGE gels, enhanced, fixed and dried. Labelled proteins were analysed by autoradiography (**Supplementary Figure 1**). Autoantibody status was determined by comparison with standard control sera. Patients with bands at 140 kDa were identified as anti-NXP2 or anti-MDA5 and differentiated through ELISA (3).

### Data analysis

Statistical analysis was undertaken using MedCalc^®^ Statistical Software version 20.015 (MedCalc Software Ltd, Ostend, Belgium). Confidence intervals (CI) are expressed at 95%. For statistics that required the prevalence of IIM, a prevalence value of 14 per 100 000 was used (12). For each ELISA assay, the area under the curve (AUC) and the Youden index was calculated. Youden’s index was used to determine if a different cut-off value in a European population could improve the overall sensitivity and specificity of the assay (13, 14). Descriptive statistics for ELISA antibody levels in each group were calculated, including the mean value and 95% confidence intervals. Cohen’s κ agreement was calculated to evaluate the correlation between antibody detection on ELISA and immunoprecipitation. Cohen’s κ agreement of <0.20, 0.21-0.4, 0.41-0.6, 0.61-0.8 and 0.81-1, demonstrating poor, fair, moderate, good and very good agreement, respectively (15).

## Results

116 sera samples with confirmed MSA antibodies were tested with ELISA, of which 109 sera samples were included after excluding samples with a co-efficient variance (CV) greater than 20%. This was compared to 246 sera samples in an HC group, of which 225 were included after samples with a CV of greater than 20% were excluded. We found a very good agreement between ELISA and immunoprecipitation, see **Table 2**.

**Table 2:** Sensitivity and specificity of autoantibodies in comparison to IP with Cohen’s K.

Autoantibody	Sensitivity (%) (95% CI)	Specificity (%) (95% CI)	*Cohen’s κ (95% CI) ± SE
**Mi2**	94.7 (74-99.9)	100 (94.3-100)	0.97 (0.9-1) **±** 0.03
**MDA5**	80 (56.3-94.3)	100 (94.2-100)	0.86 (0.7-1) **±** 0.07
**Combined ARS Antibodies**	98.6 (92.3-100)	99.1 (95.2-100)	0.98 (0.95-1)**±** 0.02
**Jo1**	100 (83.9-100)	100 (94.1-100)	1
**EJ**	100 (59–100)	100 (92.9-100)	1
**KS**	100 (15.8-100)	100 (92.8-100)	1
**PL-7**	100 (83.9-100)	100 (94–100)	1
**PL-12**	94.7 (74-99.9)	98.3 (90.8-100)	0.93 (0.8-1) **±** 0.05

*Value of Cohen’s κ and strength of agreement.

### Mi2

**Figure 1:**
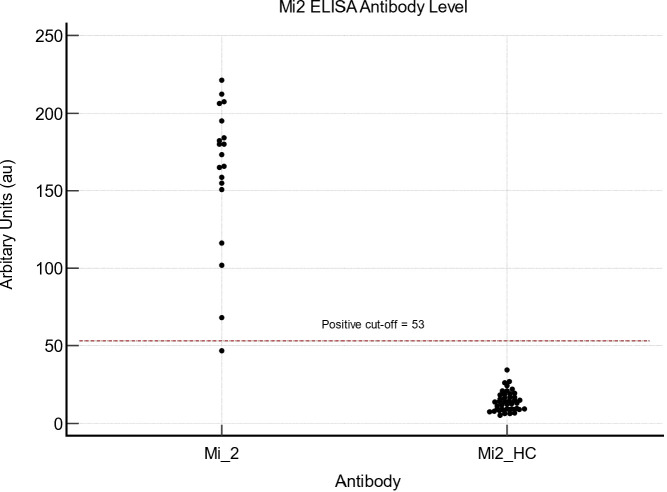
Mi2 ELISA values for 19 Mi2 and 63 healthy control samples.

Twenty anti-Mi2 sera samples previously identified through immunoprecipitation were analysed compared to sixty-three randomly selected sera samples from the HC group. In the anti-Mi2 group, ELISA successfully detected Mi2 in 18/19 sera samples. **Figure 1** demonstrates that the value of the one sera sample with a false-negative result demonstrated an antibody level of 46.8 au, 6.2 au below the pre-defined cut-off value. None of the healthy-control serum samples demonstrated a false-positive result. Compared to immunoprecipitation, sensitivity and specificity for Mi2 on ELISA were 94.7% (95% CI 74-99.9) and 100% (95% CI 94.3-100), respectively. The mean value for antibody levels for Mi2 on ELISA for the confirmed Mi2 group was 176.6 au (95% CI 155.5 – 193.1); in comparison, the mean value was 12.5 au (95% CI 10.9–14.1) in the HC group.

The AUC was 1 (95% CI 0.91 – 1, P <0.0001). The optimal cut-off point for our cohort was 34.3au, which provided a sensitivity of 100% (95% CI 82.4-100) and a specificity of 100% (95% CI 96-100).

### MDA5

**Figure 2:**
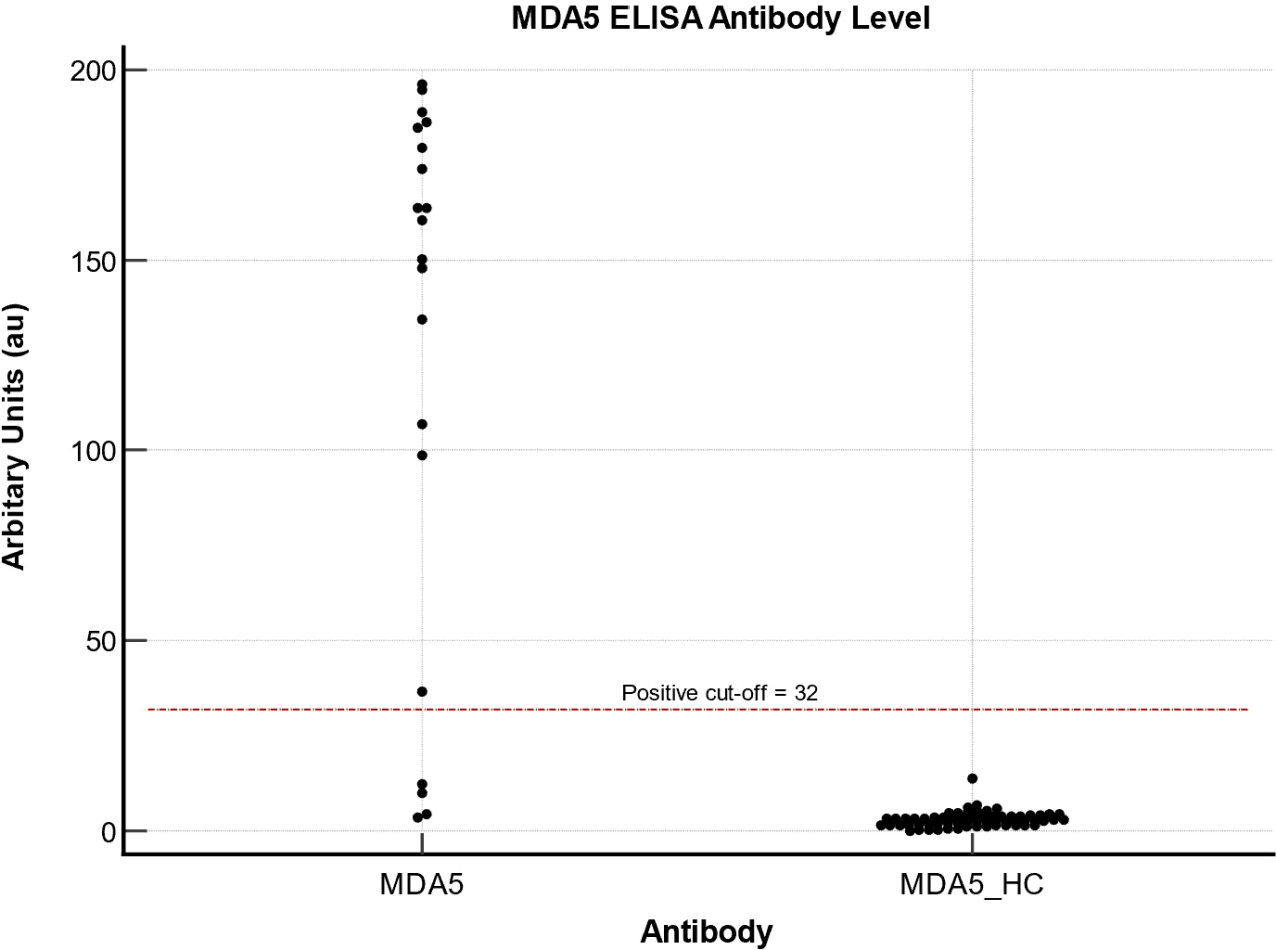
MDA5 ELISA values for 20 MDA5 and 62 healthy control samples.

Twenty anti-MDA5 positive serum samples and sixty-two autoantibody negative healthy control serum samples were analysed. As demonstrated in **Figure 2**, the MDA5 ELISA immunoassays did not detect any false-positive samples. However, four false-negative resulted in 80% sensitivity and 100% specificity. False-negative samples had antibody levels of 10 au, 4.5 au, 3.5 au and 12.4 au, well below the recommended cut-off of 32 au. The mean values for the IP confirmed anti-MDA5 serum sample group was 155.3 au (95% CI – 100-178.6); in comparison, the mean values in the HC serum sample group were 2.95 au (95% CI 2.4 – 3.5).

The AUC was 0.974 (95% CI 0.91-1, P <0.001), with sensitivity and specificity of 80% (95%CI 56.3-94.3) and 100% (95% CI 94.2-100). For our cohort of patients, the optimal cut-off for MDA5 was 6.7au, which would improve the sensitivity to 90% (95% CI 68.3-98.8) and the specificity to 98.4% (95% CI 91.3-100).

### Combined ARS antibodies

**Figure 3:**
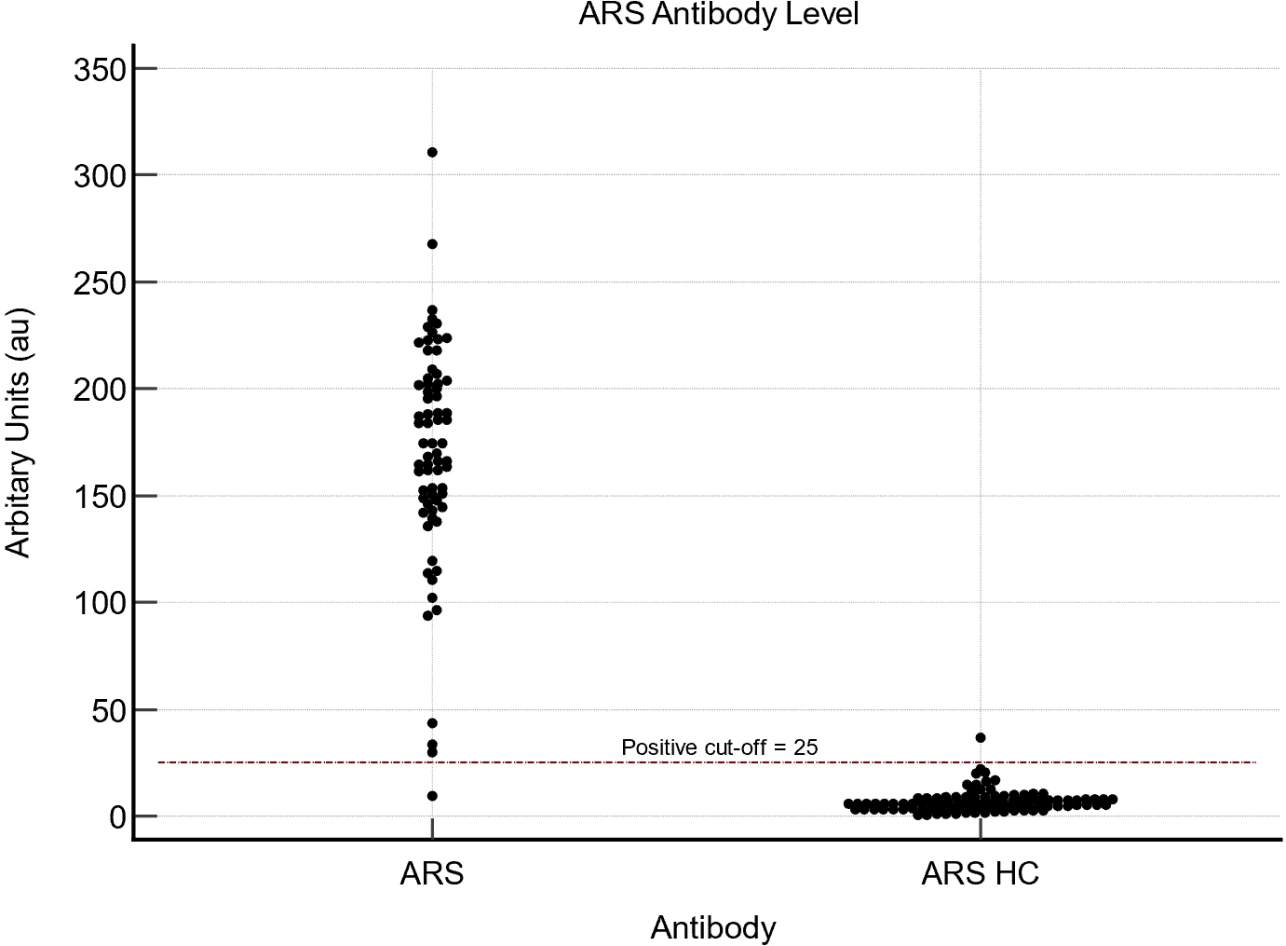
ARS ELISA values for 21 Jo1, 7 EJ, 2 Ks, 21 PL7, 19 PL12 and 117 healthy control samples.

**Figure 4:**
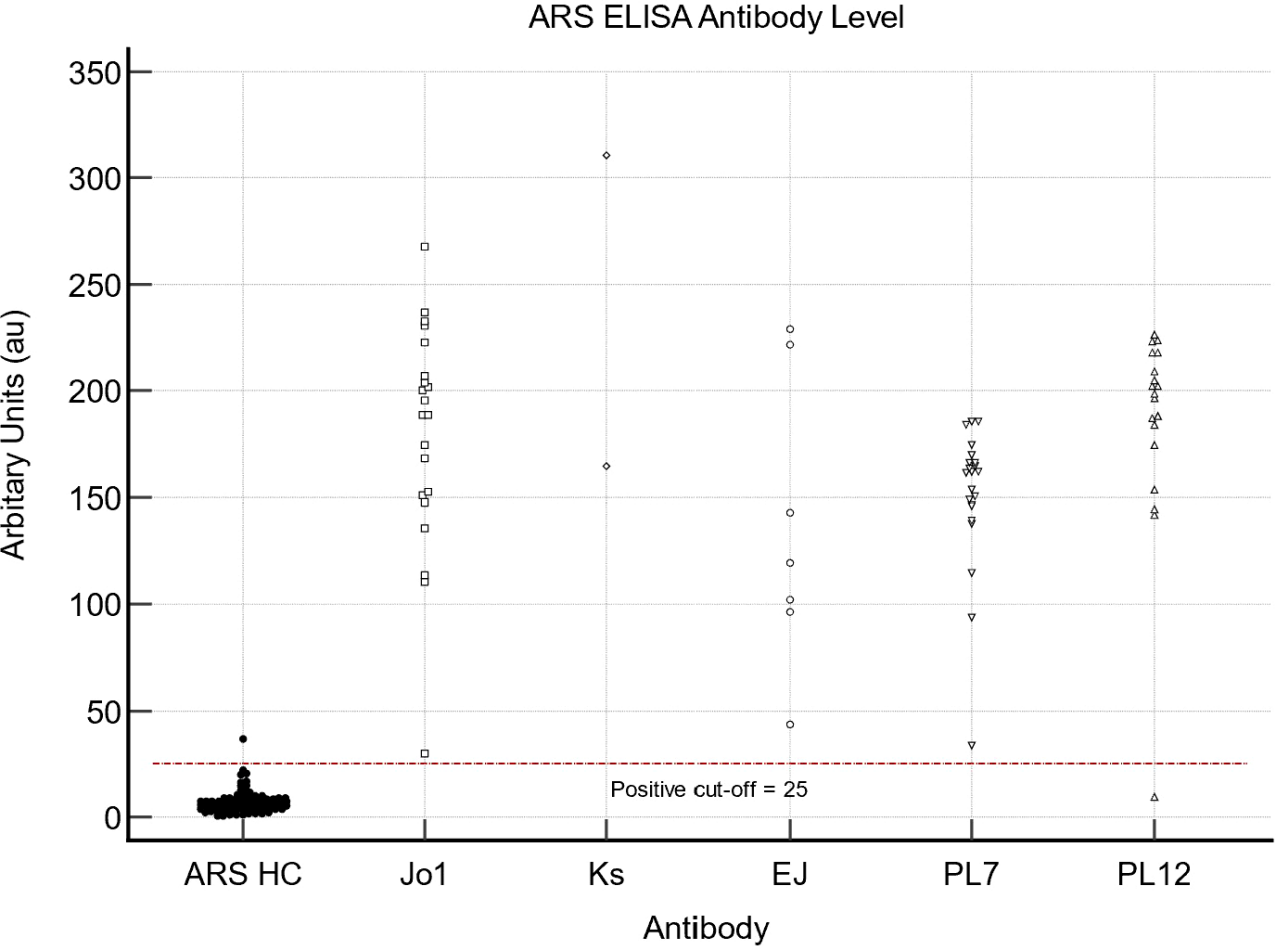
Individual ELISA values for individual ARS antibodies and HC samples.

For all ARS-antibodies, the pre-defined cut-off is 25 au. For our combined cohort of ARS antibodies (**Figure 3**), we found that the AUC was 0.997 (95% CI 0.973-1; p<0.0001). The sensitivity and specificity for the overall cohort of ARS antibodies tested were 98.6% (95% CI 92.3-100) and 99.1 (95% CI 95.2-100), respectively. In our cohort, an optimal cut-off for all ARS antibodies was determined to be 22.2 au, allowing for a sensitivity of 98.6% (95% CI 92.3-100) and specificity of 99% (95% CI 94.5-100), similar to that calculated using current cut-off values. **Figure 4** demonstrates the ELISA values for each of the individual ARS autoantibodes as well as the HC samples.

### Jo1

The mean value in the sera sample with confirmed Jo1 autoantibodies was 188.8 au (95% CI 151.96 – 204.98); in comparison, the mean value was 2.67 au (95%CI 1.46 - 5) in the HC cohort. The AUC was 1 (95% CI 0.956 – 1, p <0.0001); however, the optimal cut-off in our cohort was 20.2 au, which still provided a sensitivity and specificity of 100% (95% CI 83.9-100) and 100% (95% CI 94.1-100) respectively.

### EJ

There were no false-positive or false-negative results in the EJ cohort on ELISA, resulting in a sensitivity and specificity of 100% (95%CI 59-100) and 100% (95% 92.9-100), respectively. The mean values for the serum sample group with confirmed EJ and HC group were 119.37 au (95% CI 69.84 - 225.37) and 7.1au (95% CI 6.2 – 8.1) with an AUC of 1 (95% CI 0.937 – 1, p <0.0001). Our optimal cut-off value was 16.6 au, providing the same sensitivity of 100% (95% CI 59-100) and specificity of 100% (95% CI 92.9-100) as current cut-off values.

### KS

The sensitivity and specificity of ELISA for KS were 100% (95%CI 15.8-100) and 100% (95%CI 92.8-100). One sample, from the three available serum samples of KS had a CV value greater than 20%. Mean values for the two sera samples of Ks were 237.7au and 7.1au (95%CI 6.2-8) for the HC group. For the KS antibody, the AUC was 1 (95% CI 0.927-1, p <0.0001). Our calculated optimal cut-off value of 16.6au; provided the same sensitivity of 100% (95% CI 15.8-100) and specificity of 100% (95% CI 92.5-100), respectively.

### PL-7

All serum samples with confirmed PL-7 had positive ELISA results, and there were no false-positive results in the HC group, resulting in a sensitivity of 100% (95% CI 83.9-100) and specificity of 100% (95% CI 94-100). The mean value for the positive and HC sample groups was 161.9 au (95% CI 147.6 – 166.1) and 6.9 au (95% CI 6.1 – 7.9). The AUC was 1 (95% CI 0.96 - 1, p<0.0001). Youden’s index suggested that the optimal cut-off point for PL-7 was 16.6au, which also provides a sensitivity and specificity of 100% (95% CI 83.9 – 100) and 100% (95% CI 93.9-100), respectively.

### PL-12

Nineteen serum samples with confirmed PL-12 antibodies tested positive on ELISA. However, there was also one false-positive sample from the HC group. Sensitivity and specificity on ELISA for PL-12 were 94.7% (95% CI 74-99.9) and 93.8% (95% CI 90.8-100), respectively.Mean values for the positive serum sample and HC group were 198.5 au (95% CI 181.6 – 211.2) and 7.96 au (95% CI 6.5 – 9.4), respectively. AUC was 0.993 (95% CI 0.94 – 1, p <0.001). We calculated that an optimal cut-off value of 22.2 au improved the sensitivity and specificity to 95% (95%CI 75.1-99.9) and 100% (95% CI 93.7 – 100), respectively.

### Excluded results

After calculating the CV for the cohort of patients with confirmed MSA on IP, one serum sample from Mi2 (207.2 au), one serum sample from Jo1 (34.34 au), three serum samples from EJ (152.1 au, 46.5 au and 251.4 au), one serum sample from KS (73.7 au) and one serum sample from PL-12 (158.6 au) were excluded. All patients with excluded results in this group had absorbance values greater than cut-off points for their respective assay, meaning that no false-negative results were excluded from the final analysis.

In the healthy control group, one serum sample from the MDA5 assay (24.5 au), five samples from Jo1 (4.1 au, 5.8 au, 75.4 au, 83.4 au and 11.1 au), four samples from EJ (12.4 au, 15.8 au, 5.9 au and 78.8 au), five samples from PL-7 (10.8 au, 12.9 au, 11 au, 8.8 au, 5.7 au) and six samples from PL-12 (5.6 au, 130.4 au, 72.6 au, 6 au, 9 au and 16.1 au) had a CV greater than 20%. Of the twenty-one excluded healthy control samples, sixteen produced readings below the cut-off threshold, and five had a reading above the cut-off threshold.

### Recommended cut-off points

**Table 3** demonstrates the ideal cut-off values for our population. We determined that for Mi2, the optimal cut-off value changed from 53 au to 34.3 au, and for MDA5, the optimal cut-off changed from 32 au to 6.7 au. We also performed calculations for all ARS antibodies and each ARS antibody. For the combined ARS cohort and PL-12, the optimal cut-off changed from 25 au to 22.2 au. For the remaining ARS antibodies (Jo1, EJ, KS and PL7), the cut-off changed from 25 au to 16.6 au.

**Table 3:** New sensitivity and specificity of autoantibodies using Youden’s Index for optimal cut-off points in our cohort of patients.

Autoantibody	Current ELISA Ab cut-off (au)	New ELISA Ab cut-off (au)	AUC (95%CI)	Sensitivity 95% CI (current cut-off)	Sensitivity 95% CI (new cut-off)	Specificity 95% CI (current cut-off)	Specificity 95%CI (new cut-off)
**Mi2**	53	34.3	1 (0.91 – 1)	94.7 (74-99.9)	100 (82.4-100)	100 (94.3-100)	100 (96-100)
**MDA5**	32	6.7	0.974 (0.91-1)	80 (56.3-94.3)	90 (68.3-98.8)	100 (94.2-100)	98.4 (91.3-100)
**ARS Ab**	25	22.2	0.997 (0.973-1)	98.6 (92.3-100)	98.6 (92.3-100)	99.1 (95.2-100)	99 (94.5-100).
**Jo1**	25	20.2	1 (0.956-1)	100 (83.9-100)	100 (83.9-100)	100 (94.1-100)	100 (94.1-100)
**EJ**	25	16.6	1 (0.937-1)	100 (59-100)	100 (59-100)	100 (92.9-100)	100 (92.9-100)
**KS**	25	16.6	1 (0.927 -1	100 (15.8-100)	100 (15.8-100)	100 (92.8-100)	100 (92.5-100)
**PL-7**	25	16.6	1 (0.91-1)	100 (83.9-100	100 (83.9 – 100)	100 (94-100)	100 (93.9-100)
**PL-12**	25	22.2	0.993 (0.94-1)	94.7 (74-99.9)	95 (75.14-99.9)	98.3 (90.8-100)	100 (93.7 – 100

## Discussion

Our study suggests very good agreement between ELISA and immunoprecipitation for anti- Mi2, MDA5, Jo1, EJ, KS, PL7 and PL-12 autoantibodies. We have shown that ELISA can reliably detect MSA’s compared to reference-standard immunoprecipitation.

Being able to test the titre of MSAs by ELISA may also be able to provide the clinician with a method to measure disease activity. ELISA immunoassays are unique in providing a quantitative result with antibody levels. In a cohort of 81 patients assessed by Stone et al, Jo1 antibody levels paralleled markers of myositis disease activity (serum CK levels, muscular and articular involvement, and global MITAX). Additionally, Jo1 antibodies were not detectable in three patients during periods of disease inactivity. Longitudinal analysis of eleven patients demonstrated a significant correlation between Jo1 antibody titres and disease activity; assessed using the following parameters: muscle, joint, lung and global disease activity (16). Moreover, Jo1 antibody levels reduced by 26.6% at week 24 post-rituximab administration and 36% at week 44. Mi2 antibodies reduced by 34.8% at week 24 post-rituximab administration and 43.8% at week 44. In this study performed by Aggarwal et al., Jo1 antibody levels correlated with CK level, MMT, extra-muscular disease, physician global scores and patient global scores, while Mi2 antibody levels correlated with CK level and physician global score (17). MDA5 antibody levels also parallel patient treatment outcomes, with patients in remission having a significant decrease in antibody levels. Increases in MDA5 antibody levels are associated with an increased risk of relapse. In a study including twelve patients with confirmed MDA5, increased MDA5 levels in a patient previously in remission had a positive predictive value of 100% in successfully identifying patients with ILD relapse. Patients with a sustained positive MDA5 on ELISA were at increased risk of relapsing. Decreased MDA5 levels were associated with more extended periods of remission, and a negative MDA5 on ELISA was associated with a negative predictive value of 100% (18).

Nakashima et al. previously compared ELISA to IP in detecting anti-ARS antibodies. The sensitivity and specificity of ELISA in detecting anti-ARS antibodies were reported to be 97.1% and 99.8%, respectively (7). Sato et al. have also compared ELISA to IP in the detection of anti-MDA5, which also demonstrated high concordance, and they reported a sensitivity and specificity of 98.2% and 100%, with a positive predictive value of 100% (11). Our ARS antibodies were similar to that previously reported by Nakashima et al. (7) However, MDA5 sensitivity was significantly reduced compared to that of Sato et al. (11)

The anti-synthetase ELISA used in this study has also been developed to detect anti-OJ. This autoantibody subgroup was not assessed in our study and remains an important area for future evaluation. The detection of anti-OJ on ELISA has been reported to be problematic in the literature as there is still uncertainty regarding the best antigenic target (19, 20). Another key MSA excluded from this analysis is anti-TIF1γ. We have previously published our data on the detection of anti-TIF1γ on immunoprecipitation and ELISA. Anti-TIF1γ had a sensitivity of 97.6% and specificity of 100% on ELISA, with a false negative rate of 2% (8).

Furthermore, our study has demonstrated that the optimal cut-off points (**Table 3**) calculated using Youden’s index, compared to the recommended cut-off points, did not change the sensitivity and specificity for most ARS antibodies, including Jo1, EJ, KS and PL7. However, for PL-12, there is a slight improvement in the sensitivity and specificity. However, for Mi2 and MDA5, reducing the cut-off value would improve each assay’s sensitivity but slightly reduce the specificity of MDA5 only.

ELISA is a singleplex immunoassay, and testing for a single antibody may not be cost-effective when assessing a heterogeneous rare disease with multiple MSA’s. Multiplex immunoassays such as line blots offer increased efficiency in testing for different MSAs, but accuracy for detecting anti-TIF1γ and rarer anti-synthetase autoantibodies is reduced (3, 21). Patients being evaluated for MSA in the real world may have other autoantibodies, paraproteins or hypergammaglobulinemia that could interfere with the diagnostic performance of ELISAs (22). In addition to healthy controls, evaluating ELISA’s in other disease subgroups and patients with symptom profiles that overlap with myositis will improve our understanding of the performance of these assays in a real-world setting. ELISA immunoassays can reliably detect the presence of MSA’s in sera compared to the reference standard, immunoprecipitation. This assay type is likely to be particularly useful to screen large cohorts of patients for a single autoantibody or in select clinical scenarios with high clinical suspicion of a specific underlying MSA. The relatively high number of discrepant duplicate results is a concern in a clinical setting, as this would lead to a high number of repeat tests. However, only 5/21 discrepant sera samples without a known MSA crossed the cut-off threshold, while samples with a known MSA were still reliably detected. We performed all assays by hand, and automating the procedure will likely reduce the number of discrepant duplicate results. A unique benefit of ELISA immunoassay is that it can provide the clinician with a quantitative result with antibody levels that may reflect disease activity. Whilst the recently published British Society of Rheumatology guidelines for managing inflammatory myositis do not recommend measuring autoantibody levels to monitor disease activity based on insufficient evidence (23), this is an exciting area for future research. In the future, autoantibody titre may be used routinely in clinical practice for disease monitoring (23).

## Data availability statement

The raw data supporting the conclusions of this article will be made available by the authors, without undue reservation.

## Ethics statement

The studies involving human participants were reviewed and approved by University of Bath. The patients/participants provided their written informed consent to participate in this study.

## Author contributions

Manuscript was drafted by AL and read by SLT, NJM, BM, DL, HL, and FM. Experiments were carried out by BM, HL, FM, AL, and DL. All authors contributed to the article and approved the submitted version.

## Funding

This work was supported by funding from CureJM and the Bath Institute of Rheumatic Diseases. ELISA kits were provided by MBL. AL’s position at the Royal National Hospital of Rheumatic Diseases is part-funded by the Ken Muirden Fellowship from Arthritis Australia.

## Conflict of interest

The authors declare that the research was conducted in the absence of any commercial or financial relationships that could be construed as a potential conflict of interest.

## Publisher’s note

All claims expressed in this article are solely those of the authors and do not necessarily represent those of their affiliated organizations, or those of the publisher, the editors and the reviewers. Any product that may be evaluated in this article, or claim that may be made by its manufacturer, is not guaranteed or endorsed by the publisher.
