# Supplementary figures and images for: The use of ELISA is comparable to immunoprecipitation in the detection of selected myositis-specific autoantibodies in a European population
Source: Front Immunol. 2022 Sep 8;13:975939. doi: 10.3389/fimmu.2022.975939 (PMC9514093; doi:10.3389/fimmu.2022.975939)

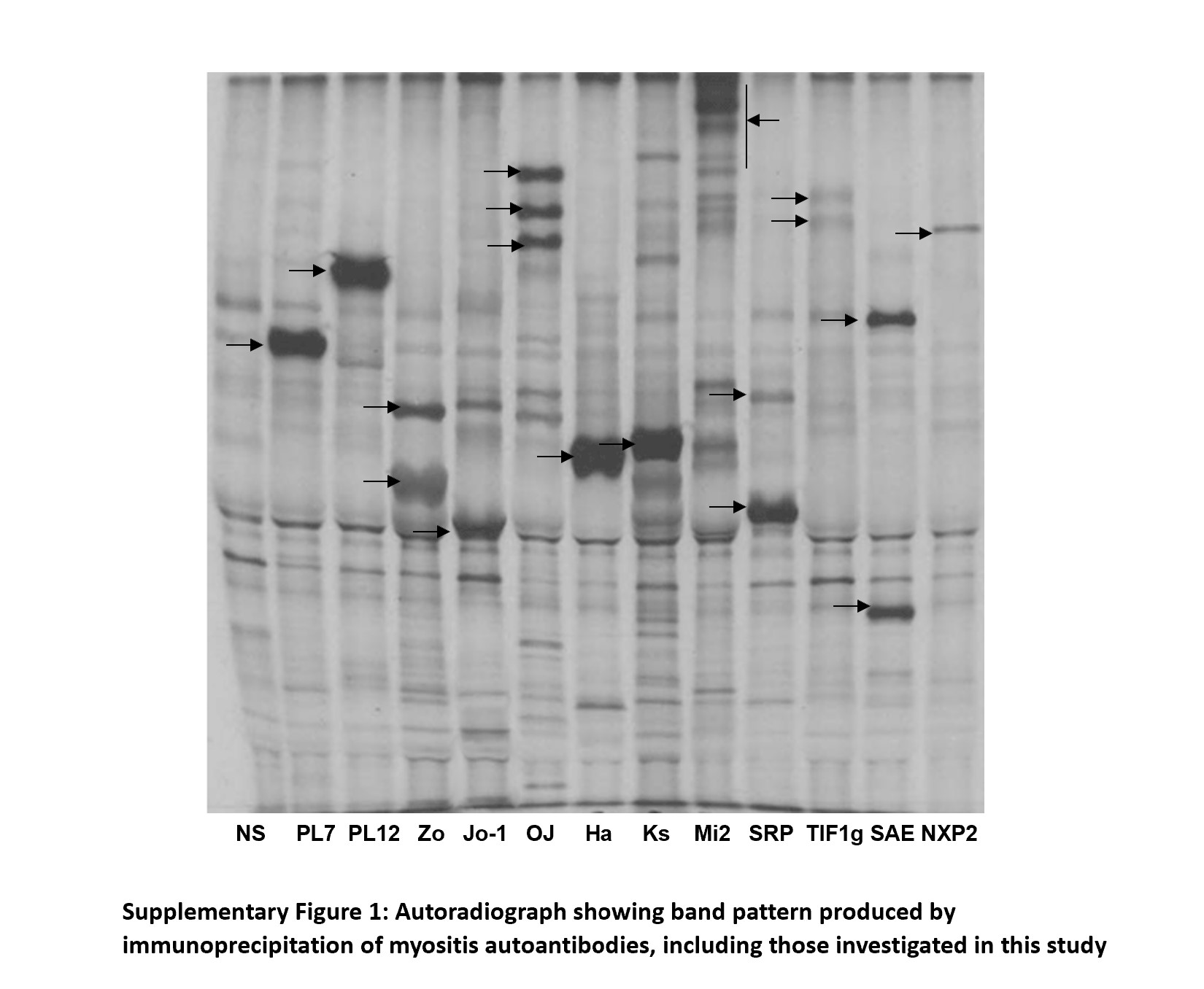

Supplement: Supplementary file 1 [file Image_1.jpeg]
